# Increasing Prevalence and High Survival Rate of Liver Transplanted Patients with NASH and PSC Cirrhosis

**DOI:** 10.34172/aim.2024.04

**Published:** 2024-01-01

**Authors:** Zohreh Khajehahmadi, Saman Nikeghbalian, Ghodratollah Roshanaei, Sina Mohagheghi

**Affiliations:** ^1^Department of Clinical Biochemistry, Faculty of Medicine, Hamadan University of Medical Sciences, Hamadan, Iran; ^2^Shiraz Organ Transplant Center, Shiraz University of Medical Sciences, Shiraz, Iran; ^3^Modeling of Noncommunicable Diseases Research Center, Hamadan University of Medical Sciences, Hamadan, Iran

**Keywords:** Cirrhosis, Hepatitis B virus, Liver transplantation, Non-alcoholic steatohepatitis, Primary sclerosing cholangitis

## Abstract

**Background:**

Epidemiological studies on liver transplant (LT) patients can provide valuable information about the etiology and trends of cirrhosis. The present study aimed to investigate the prevalence and trend of different etiologies and survival rates of LT patients at the Namazi Transplant Center in Shiraz, Iran, between 2001 and 2018.

**Methods:**

In this single-center, retrospective cohort study, the demographic and clinical characteristics of 3751 patients who underwent LT and met the study inclusion criteria, including age, gender, blood group, body mass index, model for end-stage liver disease (MELD) score, cause of cirrhosis, and diabetes, were extracted from patients’ physical or electronic medical records between 2001 and 2018.

**Results:**

The MELD scores of LT patients with primary sclerosing cholangitis (PSC), hepatitis B virus (HBV), and non-alcoholic steatohepatitis (NASH) cirrhosis significantly decreased over the study period (*P*<0.001). Among the LT patients, HBV infection had the highest frequency (21.09%), followed by cryptogenic (17.33%) and PSC (17.22%). The proportion of patients with PSC and NASH (both *P*<0.001) cirrhosis was significantly increased, so that PSC cirrhosis (2016: 19.4%, 2018: 18.8%) surpassed HBV (2016: 18.4%, 2018: 13.5%), autoimmune hepatitis (2016: 11.7%, 2018: 12.7%), and cryptogenic cirrhosis (2016: 16.1%, 2018:14%) as the leading indication for LT from 2016 to the end of the study period. Fortunately, these patients had a better survival rate than other common diseases (HR: 0.53, CI: 0.43‒0.66; *P*<0.001).

**Conclusion:**

The proportion of NASH and PSC cirrhosis significantly increased during the 18 years of study. However, these patients had an improved survival rate. Therefore, health organizations should pay more attention to non-communicable diseases, especially fatty liver disease and cholangitis.

## Introduction

 Despite major efforts over the past decade in the prevention, diagnosis, and treatment of liver diseases, it remains one of the leading causes of death in low-income and lower-middle-income countries.^1^ There are several known and unknown causes of liver injury and end-stage liver disease (ESLD). Hepatocellular carcinoma and cirrhosis. Chronic hepatitis B virus (HBV) and hepatitis C virus (HCV) infections, excessive alcohol consumption, autoimmune diseases, and metabolic syndrome secondary to obesity are major causes of liver disease.^2^ Patients with liver cirrhosis without any known etiology are referred to as those with cryptogenic cirrhosis.^3^ These patients comprised a considerable proportion of those with cirrhosis.

 Unfortunately, liver transplantation (LT) is the only effective treatment for patients with ESLD.^4^ In several previous studies, the epidemiology of patients with liver cirrhosis has been studied, mainly using the data of patients on the waitlist for LT.^5-7^ However, among these patients, those who underwent LT have more severe disease than others and usually have a worsened model for end-stage liver disease (MELD) score. Hence, there could be some differences between liver transplanted patients and the general population of patients with ESLD in terms of their prevalence trend. Therefore, epidemiological studies of liver-transplanted patients can provide valuable information about the etiology and trends of cirrhosis from a different perspective. It has been reported that HBV and/or HCV cirrhosis, cryptogenic cirrhosis, autoimmune hepatitis (AIH) cirrhosis, and alcoholic cirrhosis (in some regions of the world) are the main causes of cirrhosis.^6^ However, it is unclear whether these etiologies are the main causes of cirrhosis among liver‐transplanted patients. In addition, the proportion of some cirrhosis etiologies, such as non-alcoholic steatohepatitis (NASH) cirrhosis, has increased over time in the waitlist for LT, while the proportion of some cirrhosis etiologies, such as HBV cirrhosis, has decreased worldwide.^8-10^ Similar to the frequency of patients with ESLD, the trend in the proportion of different etiologies on the waitlist for LT is not necessarily in accordance with the trend in the proportion of different etiologies in liver‐transplanted patients. The other important issue is the survival rate of LT patients. If a disease has an increasing prevalence and, on the other hand, a lower survival rate, it becomes much more important. Therefore, this retrospective observational study seeks to investigate the demographic characteristics and survival rates of patients undergoing LT at the Namazi Transplant Center, the main LT center in Iran, and to describe the changes in the epidemiology of these patients between 2001 and 2018.

## Materials and Methods

###  Study Design

 In this single-center, retrospective cohort study, we extracted and analyzed the demographic characteristics of patients who underwent LT between 2001 and 2018 at Namazi Transplant Center, affiliated with Shiraz University of Medical Sciences, Shiraz, Iran. From a total of 4835 consecutive LT patients at this center between 2001 and 2018, 3751 patients met the study inclusion criteria. All patients aged > 18 years who underwent LT at Namazi Transplant Center, irrespective of etiology, were included in this study, and all organ donors were brain-death cases. Before LT, the cirrhosis of all patients and its etiologies were diagnosed using clinical symptoms, radiological assessments, and laboratory findings, and the diagnosis was confirmed by histopathological examination of the explanted liver. Patients with incomplete demographic and clinical data from their medical records were excluded from this study.

 Demographic and clinical data, including age, gender, blood group, body mass index (BMI), MELD score, date of transplantation, cause of cirrhosis, and presence of diabetes for each patient, were extracted from the patients’ physical or electronic medical records. The electronic medical records of some patients were checked with their physical medical records as a double-checking mechanism.

 Cirrhosis etiologies with frequencies of more than 40 cases were considered the main causes of cirrhosis, which comprised ten etiologies, including acute liver failure, alcoholism, AIH, Budd-Chiari, HBV infection, HCV infection, NASH, primary biliary cholangitis (PBC), primary sclerosing cholangitis (PSC), and Wilson. Patients with more than one etiology were categorized as “mixed etiology”, and those with uncommon etiology, such as Alpha-1 antitrypsin deficiency, amyloidosis, Caroli’s disease, colorectal metastasis, Crigler-Najjar, fibromatosis, hemangioma, and the like, were categorized as the “other group.” The frequency of these etiologies was lower than 15 patients. In addition, all cirrhotic patients with unknown etiology were categorized as the “cryptogenic group.”

###  Statistical Analysis

 All obtained data were analyzed using IBM SPSS, version 16.0 (SPSS, Inc., Chicago, IL). Kaplan-Meier methods and the log-rank test were used to assess overall survival. Continuous variables are presented as means ± standard deviations (SD) and compared using the Student’s *t*-test, and categorical variables are expressed as frequencies and proportions and compared using the chi-square test. Statistical significance was set at *P* < 0.05.

## Results

###  Demographic Characteristics

 From a total of 4835 consecutive LT patients at Namazi Transplant Center between 2001 and 2018, 3751 patients met the study inclusion criteria, and their demographic data were extracted and summarized with reference to the underlying etiology in Table 1. Patients with AIH (mean ± SD: 34.4 ± 11.8 years) and Budd-Chiari (mean ± SD: 33.9 ± 8.3 years) cirrhosis had the lowest mean ( ± SD) age, while those with NASH cirrhosis (mean ± SD: 54.4 ± 8 years) had the highest mean ( ± SD) age. The proportion of male patients with all etiologies was higher than that of females, with the exception of AIH cirrhosis. The mean MELD score of patients with PSC (mean ± SD: 19.4 ± 6.0) and Budd-Chiari cirrhosis (mean ± SD: 19.7 ± 4.7) was lower than that of patients with other etiologies. Patients with PSC (mean ± SD: 22.5 ± 3.7) and Wilson disease (mean ± SD: 22.7 ± 3.8) had the lowest BMI values, whereas those with NASH cirrhosis (mean ± SD: 28.5 ± 4.8) had the highest BMI values in comparison to other etiologies. Patients with NASH cirrhosis had the highest proportion of diabetes (25.2%), followed by alcoholic (9.5%) and HBV (8.6%) cirrhosis. However, Wilson (0.6%) and Budd-Chiari (0.8%) had the lowest proportion compared to the other age groups. Finally, the blood group distribution was similar among different etiologies.

**Table 1:** Demographic Characteristics and Median Overall Survival of Liver Transplanted Patients with Reference to Underlying Etiology

	**Total**	**Acute Liver Failure**	**Alcoholic**	**AIH**	**Budd-Chiari**	**Cryptogenic**	**HBV**	**HCV**	**NASH**	**PBC**	**PSC**	**Wilson**	**HCC**	**Mixed**	**Other**
Subjects (Number)	3751	60	42	516	120	650	791	125	147	55	646	177	73	132	217
Male gender (% male)	2365 (63)	25 (41.7)	42 (100)	172 (33.3)	55 (45.8)	441 (67.8)	654 (82.7)	89 (71.2)	109 (74.1)	8 (14.5)	406 (62.8)	115 (65)	57 (78.1)	70 (53)	122 (56.2)
Age (mean ± SD)	42.1 ± 13.4	37.1 ± 12.6	47.1 ± 11.3	34.4 ± 11.8	33.9 ± 8.3	44.5 ± 13.8	50.1 ± 9.7	51.3 ± 8.3	54.4 ± 8	45.9 ± 9.8	37.2 ± 10.9	27.1 ± 7.2	46.5 ± 14.3	42.1 ± 12.5	36.9 ± 13.1
MELD (mean ± SD)	21.5 ± 6.5	35.1 ± 6.3	26.5 ± 6.1	22.6 ± 5.9	19.7 ± 4.7	22.1 ± 5.5	21.5 ± 5.7	21.2 ± 5.5	21.5 ± 6.3	21.3 ± 7.1	19.4 ± 6.0	21.6 ± 7.2	19.7 ± 7.2	21.8 ± 7.2	19.9 ± 9.4
BMI (mean ± SD)	24.3 ± 4.4	24.3 ± 4.5	24.3 ± 3.9	23.9 ± 4.4	23.1 ± 4.3	25.2 ± 4.2	25.2 ± 4.2	25.0 ± 3.4	28.5 ± 4.8	24.2 ± 4.3	22.5 ± 3.7	22.7 ± 3.8	24.0 ± 4.1	24.4 ± 4.3	23.4 ± 4.8
Diabetics (% within etiology)	218 (5.8)	3 (5)	4 (9.5)	19 (3.7)	1 (0.8)	37 (5.8)	68 (8.6)	10 (8)	37 (25.2)	3 (5.5)	14 (2.2)	1 (0.6)	5 (6.8)	9 (6.8)	7 (3.2)
Blood group (% within etiology)															
A	30.1	40.7	25.6	32.1	36.7	29.2	30.7	25.8	28.1	31.5	31	29.5	31.5	22.9	26.3
B	24.9	16.9	28.2	23.8	22.5	27.5	24.2	20.2	27.4	20.4	25.1	23.9	19.2	29	27.2
AB	8.3	6.8	5.1	6.1	8.3	8.2	6.6	15.3	8.9	9.3	9.7	8	8.2	11.5	10.3
O	36.7	35.6	41	38	32.5	35.1	38.5	38.7	35.6	38.9	34.3	38.6	41.1	36.6	36.2
Median OS (month)		-	-	224	-	223	224	186	-	239	225	-	-	-	200
Quarter 1 OS (month)		11	50	196	164	150	107	32	31	65	171	177	10	100	6
Hazard ratio (CI)		1.09 (0.7-1.69)	0.67 (0.34-1.29)	1.46 (0.36-2.87)	0.76 (0.52-1.13)	0.68 (0.56-0.82)	0.74 (0.62-0.88)	1.03 (0.75-1.42)	0.78 (0.6-1.28)	0.47 (0.24-0.91)	0.53 (0.43-0.66)	0.64 (0.49-0.84)	1.49 (1.17-1.91)	-	-

*Note*. AIH, autoimmune hepatitis; HBV, hepatitis B virus; HCV, hepatitis C virus; NASH, nonalcoholic steatohepatitis; PBC, primary biliary cholangitis; PSC, primary sclerosing cholangitis; HCC, hepatocellular carcinoma; MELD, model for end-stage liver disease; BMI, body mass index; OS, overall survival; HR, hazard ratio; CI, confidence interval.

###  Demographic Characteristics in Different Gender and Diabetic Patients

**Figure 1:**
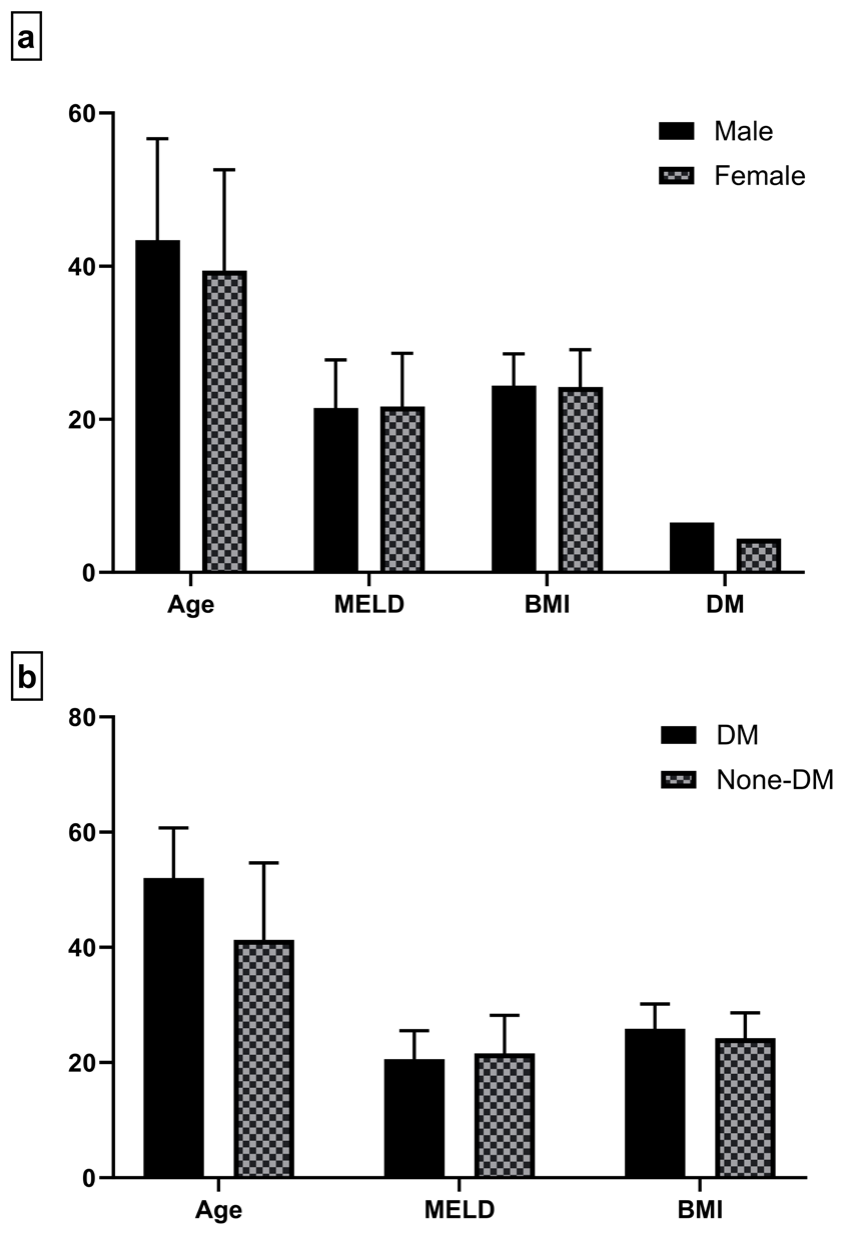
Demographic Characteristics of Liver Transplant Patients in **(a)** Two Different Genders and **(b)** Those with or Without Diabetes. *Note*. MELD: Model for end-stage liver disease; BMI: Body mass index; DM: Diabetes mellitus

 The mean ( ± SD) age of male patients (43.39 ± 13.25 years) was significantly higher than that of females (39.43 ± 13.17 years) (*P* < 0.001). However, there was no significant difference in the MELD score and BMI value between males and females, and the blood group distribution was similar between genders. In addition, the proportion of male patients with diabetes (6.5%) was higher than that of female patients with diabetes (4.4%) (Figure 1a). In contrast, the MELD and BMI values of LT patients with diabetes were similar to those of nondiabetic patients. However, the mean ( ± SD) age of diabetic patients (52.05 ± 8.67 years) was significantly higher than that of nondiabetics (41.32 ± 13.35 years) (*P* < 0.001, Figure 1b).

###  Trends in the Distribution of Demographic Characteristics and Etiologies in Each Etiology From 2001 to 2018

**Figure 2:**
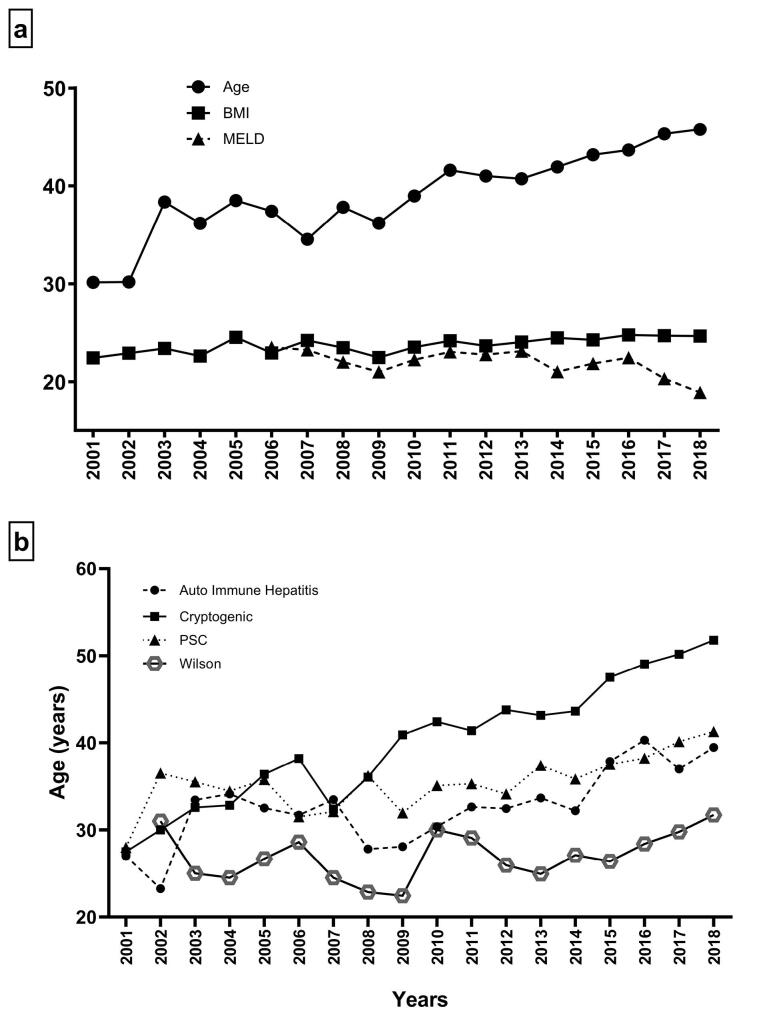
**(a)** Changes in the Age, BMI, and MELD Score of Transplanted Patients and **(b)** Trend in the Mean Age of Transplanted Patients with Autoimmune Hepatitis, Cryptogenic, PSC, and Wilson Cirrhosis Between 2001 and 2018 in Namazi Transplant Center in Iran. *Note*. BMI: Body mass index; MELD: Model for end-stage liver disease; PSC: Primary sclerosing cholangitis

 Among the demographic characteristics of all transplants, only the mean age of patients significantly increased over the study period, while the other variables did not change (Figure 2a). Further, the trend in the demographic characteristics of all etiologies was studied, and our results revealed that, with some exceptions, there was no significant trend in the demographic characteristics of all etiologies. The exceptions were age, which significantly increased in patients with HBV (*P* < 0.001), AIH (*P* < 0.001), and PSC (*P* = 0.003), as well as cryptogenic (*P* < 0.001) cirrhosis (Figure 2b) and MELD score, which significantly decreased in patients with PSC, HBV, and NASH cirrhosis (all *P* < 0.001).

**Figure 3:**
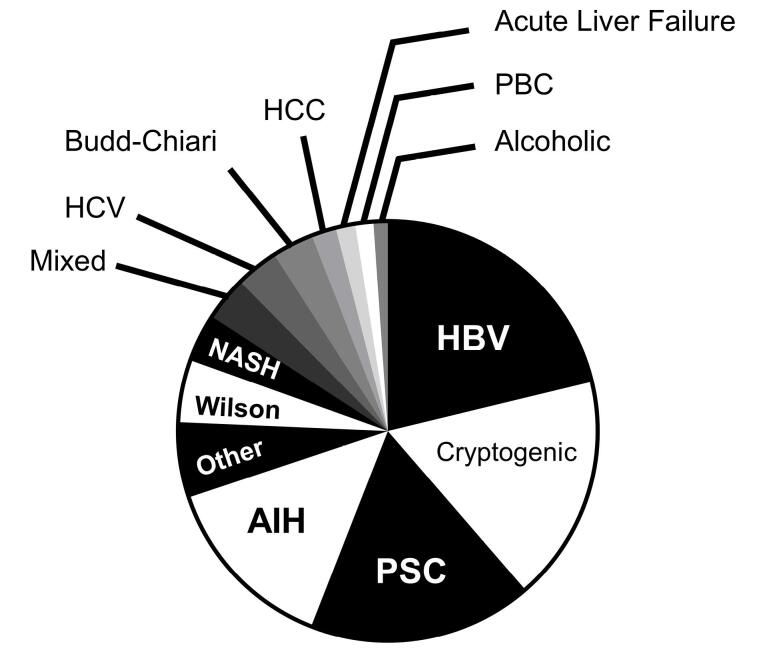
Proportion of Transplant Patients Between 2001 and 2018 in the Namazi Transplant Center, Iran. *Note*. HBV: Hepatitis B virus; PSC: Primary sclerosing cholangitis; AIH: Autoimmune hepatitis; NASH: Nonalcoholic steatohepatitis; HCV: Hepatitis C virus; HCC: Hepatocellular carcinoma; PBC: Primary biliary cholangitis

**Figure 4:**
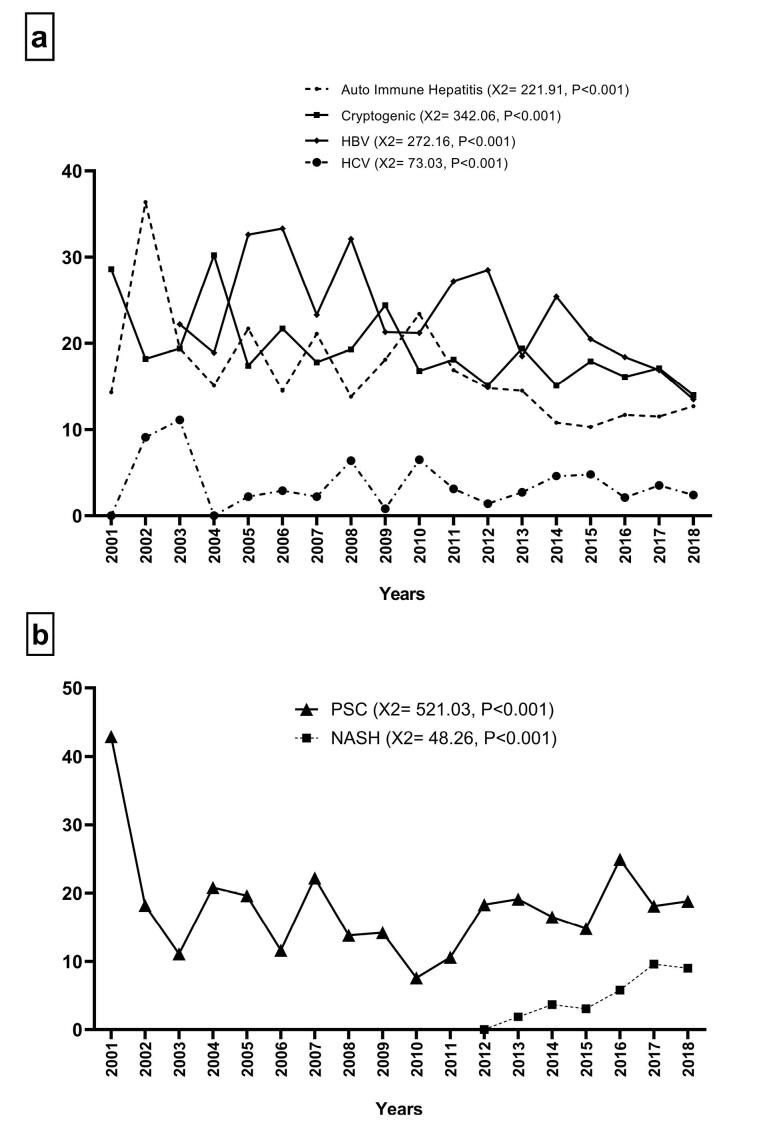
Trend in the Proportion of Transplanted Patients **(a)** With Autoimmune Hepatitis, Cryptogenic, HBV, and HCV Cirrhosis and **(b)** With PSC and NASH Cirrhosis Between 2001 and 2018 in Namazi Transplant Center, Iran. *Note*. HBV: Hepatitis B virus; HCV: Hepatitis C virus; PSC: Primary sclerosing cholangitis; NASH: Nonalcoholic steatohepatitis

 Among LT patients, HBV infection had the highest frequency (n = 791–21.09%), followed by cryptogenic (n = 650–17.33%), PSC (n = 646–17.22%), and AIH (n = 516–13.76%) cirrhosis (Figure 3). During the study period, the proportion of patients with HBV (χ^2^ = 272.16, *P* < 0.001), HCV (χ^2^ = 73.03, *P* < 0.001), AIH (χ^2^ = 221.91, *P* < 0.001), and cryptogenic (χ^2^ = 342.06, *P*< 0.001) cirrhosis significantly decreased (Figure 4a), while the proportion of patients with PSC (χ^2^ = 521.03, *P* < 0.001) and NASH (χ^2^ = 48.26, *P*< 0.001) cirrhosis represented a significant increase (Figure 4b). However, the proportion of patients with other etiologies did not change significantly during the study period. Due to these trends, we investigated the proportion of each etiology in each year of the study period. Due to the low frequency of LT before 2008, we considered the proportion of each etiology in each year between 2008 and 2018 (Table 2). Our results demonstrated that HBV cirrhosis was the leading indication for LT between 2008 and 2015, with the exception of 2009, 2010, and 2013. Cryptogenic cirrhosis was the leading indication for LT in 2009 and 2013, and AIH cirrhosis was the leading indication for LT in 2010. Conversely, PSC cirrhosis surpassed HBV, AIH, and cryptogenic cirrhosis as leading indications for LT between 2016 and 2018. Regarding the NASH cirrhosis trend, it is predicted that NASH will surpass PSC as a leading indication for LT in the near future.

**Table 2:** The Proportion of Liver Transplanted Patients With Different Cirrhosis Etiologies in 11 Years Between 2008 and 2018

	**2008** **%**	**2009** **%**	**2010** **%**	**2011** **%**	**2012** **%**	**2013** **%**	**2014** **%**	**2015** **%**	**2016** **%**	**2017** **%**	**2018** **%**
HBV	32.1%*	21.3%	21.2%	27.2%*	28.5%*	18.5%	25.4%*	20.5%*	18.4%	16.9%	13.5%
Cryptogenic	19.3%	24.4%*	16.8%	18.1%	15.1%	19.4%*	15.1%	17.9%	16.1%	17.1%	14.0%
AIH	13.8%	18.1%	23.4%*	16.9%	14.8%	14.5%	10.8%	10.3%	11.7%	11.5%	12.7%
PSC	13.8%	14.2%	7.6%	10.6%	18.3%	19.1%	16.5%	14.8%	24.9%*	18.1%*	18.8%*
NASH	0.0%	0.0%	0.0%	0.0%	0.0%	1.9%	3.7%	3.1%	5.8%	9.6%	9.0%
PBC	0.9%	0.8%	2.7%	0.8%	1.8%	1.3%	1.4%	1.9%	0.5%	1.0%	2.1%
HCV	6.4%	0.8%	6.5%	3.1%	1.4%	2.7%	4.6%	4.8%	2.1%	3.5%	2.4%
Wilson	6.4%	7.1%	4.3%	5.1%	6.0%	4.8%	5.4%	5.0%	3.0%	2.7%	4.5%
Alcoholic	1.8%	2.4%	1.6%	2.4%	1.1%	1.1%	0.9%	0.7%	1.2%	0.8%	0.8%
Budd-Chiari	2.8%	2.4%	5.4%	2.8%	2.5%	3.5%	5.7%	3.6%	3.0%	3.3%	2.4%
Mixed	0.0%	1.6%	3.3%	4.3%	4.6%	4.0%	3.1%	2.6%	2.8%	4.4%	5.3%
other	2.8%	5.5%	4.3%	7.1%	4.2%	5.9%	4.3%	9.8%	4.9%	5.8%	9.5%

*Note*. ^*^The highest proportion in the year; HBV, hepatitis B virus; AIH, autoimmune hepatitis; PSC, primary sclerosing cholangitis; NASH, nonalcoholic steatohepatitis; PBC, Primary biliary cholangitis; HCV, hepatitis C virus.

###  Survival Rates of Liver Transplant Patients

 The median overall survival of LT patients is provided in Table 1. The median overall survival of patients was 224 months. There were significant differences between LT patients with different causes of disease (*P*< 0.001). In parallel with the increasing frequency of PSC cirrhosis, patients with PBC and PSC had a better median overall survival than other patients (239 and 225 months, respectively). Furthermore, among the common causes of liver diseases, NASH cirrhosis had a better survival rate than others. The patients with HCV cirrhosis had the lowest median overall survival (186 months).

## Discussion

 The data presented in the present study were collected from the largest liver transplant center in the world over 18 years; therefore, it provides valuable information about LT patients.^11^ In this single-center retrospective study, it was found that LT patients with NASH cirrhosis had the highest mean age, BMI, and diabetes, while those with Budd-Chiari cirrhosis had the lowest mean age, MELD, and diabetes. These findings suggest that, unlike NASH cirrhosis, Budd-Chiari cirrhosis did not correlate with metabolic disease and progressed rapidly to cirrhosis; thus, the MELD score could not properly show the disease severity. AIH cirrhosis is the only disease whose proportion in females is higher than in males, which is likely due to the higher prevalence of autoimmune diseases in women than in men.^12^ These patients had a low mean age, indicating rapid progression to cirrhosis.^13^ Similar to Budd-Chiari cirrhosis, LT patients with PSC cirrhosis had low MELD scores and BMI values. Regarding the clinical findings of the patients and performing transplantation for them, these findings declare that the MELD score poorly reflects the disease severity in patients with PSC and Budd-Chiari cirrhosis. Unlike our study results, it has been reported that African-American patients tend to be younger and have higher MELD scores at the time of LT, suggesting a more aggressive disease phenotype.^14^ Therefore, PSC cirrhosis may have different disease phenotypes in different geographical regions.

 A comparison of demographic characteristics between male cirrhotic patients and females revealed that the mean age of males and the proportion of diabetics in male patients were higher than those in females. The lower mean age of females may be due to the high proportion of AIH cirrhosis, its rapid progression, and its higher proportion in females. Unexpectedly, the mean age of the diabetic patients was significantly higher than that of the nondiabetic patients. Further investigation is required to clarify the association between age and diabetes in patients with cirrhosis.

 Analysis of demographic characteristics over the study period showed that only the age of LT patients significantly increased, and more investigations demonstrated that this increase was mainly related to the increase in the age of patients with HBV, AIH, PSC, and cryptogenic cirrhosis. Similar to our findings, it has been reported that the age of patients with liver disease increased in the USA between 1988 and 2016.^15^ Another study indicated that the proportion of patients with newly diagnosed cirrhosis aged ≥ 65 years increased in Indiana between 2004 and 2014.^16^ In contrast, the MELD score of LT patients with PSC, HBV, and NASH cirrhosis significantly decreased over the study period. Although the increase in the mean age of the patients is a positive point because of the high prevalence of the aforementioned etiologies, the decrease in the MELD score of LT patients indicates a decrease in the reliability of the MELD score to determine priority for LT for the related etiologies. However, it should be noted that these changes in demographic characteristics over the study period may have resulted from an increase in the absolute number of LT patients.

 Until 2015, HBV cirrhosis was the most common indication for LT in most years of the study period, and PSC cirrhosis surpassed HBV cirrhosis as the leading indication for LT from 2016 to 2018. There are some similarities and differences between our findings and those of similar studies. One study reported that HCV cirrhosis was the most common indication for LT in Saudi Arabia until 2016, after which it was surpassed by NASH cirrhosis. However, it has been shown that PSC and AIH cirrhosis had a low proportion among patients who underwent LT in the country.^17^ In contrast, another study reported that the proportion of alcoholic cirrhosis, the third leading indication for LT, and NASH cirrhosis increased between 2000 and 2015, while the proportion of HCV cirrhosis decreased during the study period in the United States.^18^ In accordance with our study, an analysis of data collected from UK and US national liver transplant registries between 1995 and 2014 declared that PSC is the leading indication for LT among patients with autoimmune liver diseases, including PSC, PBC, and AIH.^19^ Therefore, the common denominator of all these studies was an increase in the proportion of LT patients with NASH cirrhosis and a decrease in the proportion of those with HCV cirrhosis over time. The reason for the decrease in the HCV cirrhosis proportion may be the widespread use of improved therapy for HCV, including direct-acting antiviral agents.^20^ In contrast, while alcoholic cirrhosis is one of the major indications for LT in most European and American countries, this etiology has a low frequency in Islamic countries due to religious beliefs and legal bans on alcohol consumption.^21,22^ On the other hand, the increase in the proportion of NASH cirrhosis is mainly due to poor lifestyles, which has led to an increase in the prevalence of NASH. Finally, it should be noted that PSC cirrhosis, as an autoimmune disease, was the leading indication for LT in the last three years of the study period at Namazi Transplant Center, and it is a warning to healthcare providers to find and control possible predisposing factors, especially environmental factors that result in PSC cirrhosis. However, the promising point is that LT patients with cholestatic diseases, including PSC and PBC cirrhosis, had the best survival rate compared to the other common diseases. In line with our findings, in another liver transplant center, it has been found that patients with biliary and metabolic diseases had better survival rates than patients with other etiologies.^23^

## Conclusion

 In conclusion, in this single-center, retrospective cohort study, LT patients with the male gender, NASH cirrhosis, and diabetes were significantly older than LT patients with the female gender, other etiologies, and no diabetes, respectively. AIH cirrhosis was more prevalent in women than in men, and these patients had the lowest mean age. The mean age of the LT patients significantly increased over the study period, especially in patients with HBV, AIH, PSC, and cryptogenic cirrhosis. In addition, the MELD scores of LT patients with PSC, HBV, and NASH cirrhosis significantly decreased between 2001 and 2018. Finally, in addition to NASH cirrhosis, the proportion of PSC cirrhosis significantly increased during the 18 years of study, which became the leading indication for LT in the last three years of the study period. However, LT patients with NASH and PSC cirrhosis had a better survival rate than other common diseases. Therefore, it is recommended that healthcare providers in Iran consider changes in the demographic characteristics of liver-transplanted patients in their decisions.
